# Tetra­aqua­bis(2-oxo-1,2-dihydro­pyridine-5-sulfonato-κ*O* ^2^)zinc(II)

**DOI:** 10.1107/S1600536809039774

**Published:** 2009-10-07

**Authors:** Zhi-Biao Zhu, Shan Gao, Seik Weng Ng

**Affiliations:** a College of Chemistry and Materials Science, Heilongjiang University, Harbin 150080, People’s Republic of China; b Department of Chemistry, University of Malaya, 50603 Kuala Lumpur, Malaysia

## Abstract

The metal atom in the title compound, [Zn(C_5_H_4_NO_4_S)_2_(H_2_O)_4_], lies on a center of inversion and is linked to the anionic ligand through the carbonyl O atom. In the crystal structure, the 2-oxo-1,2-dihydro­pyridine-5-sulfon­ate ligand inter­acts with other mol­ecules through N—H⋯O and O—H⋯O hydrogen bonds, forming a three-dimensional network structure.

## Related literature

For the crystal structure of another zwitterionic tetra­aqua­bis(amide)–metal^II^ complex, see: Gao *et al.* (2004 ▶).
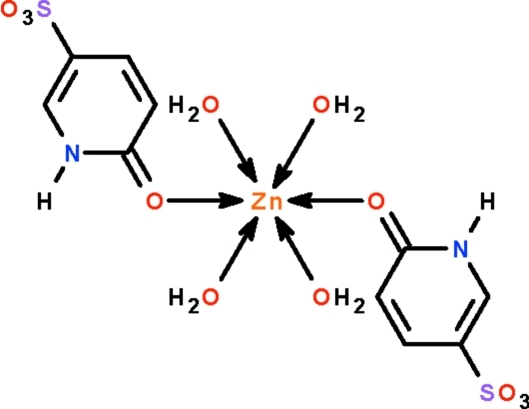


## Experimental

### 

#### Crystal data

[Zn(C_5_H_4_NO_4_S)_2_(H_2_O)_4_]
*M* *_r_* = 485.74
Monoclinic, 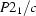

*a* = 6.7701 (2) Å
*b* = 13.9725 (5) Å
*c* = 10.0343 (3) Å
β = 115.331 (2)°
*V* = 857.93 (5) Å^3^
*Z* = 2
Mo *K*α radiation
μ = 1.74 mm^−1^
*T* = 293 K
0.21 × 0.16 × 0.16 mm

#### Data collection

Rigaku R-AXIS RAPID IP diffractometer
Absorption correction: multi-scan (*ABSCOR*; Higashi, 1995 ▶) *T*_min_ = 0.711, *T*_max_ = 0.768
8224 measured reflections
1951 independent reflections
1866 reflections with *I* > 2σ(*I*)
*R*_int_ = 0.021

#### Refinement

*R*[*F* ^2^ > 2σ(*F* ^2^)] = 0.023
*wR*(*F* ^2^) = 0.066
*S* = 1.06
1951 reflections
144 parameters
5 restraints
H atoms treated by a mixture of independent and constrained refinement
Δρ_max_ = 0.37 e Å^−3^
Δρ_min_ = −0.39 e Å^−3^

### 

Data collection: *RAPID-AUTO* (Rigaku, 1998 ▶); cell refinement: *RAPID-AUTO*; data reduction: *CrystalClear* (Rigaku/MSC, 2002 ▶); program(s) used to solve structure: *SHELXS97* (Sheldrick, 2008 ▶); program(s) used to refine structure: *SHELXL97* (Sheldrick, 2008 ▶); molecular graphics: *X-SEED* (Barbour, 2001 ▶); software used to prepare material for publication: *publCIF* (Westrip, 2009 ▶).

## Supplementary Material

Crystal structure: contains datablocks global, I. DOI: 10.1107/S1600536809039774/xu2623sup1.cif

Structure factors: contains datablocks I. DOI: 10.1107/S1600536809039774/xu2623Isup2.hkl

Additional supplementary materials:  crystallographic information; 3D view; checkCIF report

## Figures and Tables

**Table 1:** Hydrogen-bond geometry (Å, °)

*D*—H⋯*A*	*D*—H	H⋯*A*	*D*⋯*A*	*D*—H⋯*A*
N1—H1⋯O2^i^	0.85 (1)	1.99 (1)	2.790 (2)	157 (2)
O1w—H11⋯O2^ii^	0.84 (1)	1.98 (1)	2.809 (2)	171 (2)
O1w—H12⋯O3^iii^	0.84 (1)	1.93 (1)	2.767 (2)	172 (3)
O2w—H21⋯O3^iv^	0.83 (1)	2.13 (1)	2.926 (2)	160 (3)
O2w—H22⋯O4^v^	0.84 (1)	1.93 (1)	2.765 (2)	174 (3)

Symmetry codes: (i) 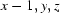; (ii) 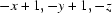; (iii) 
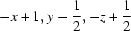
; (iv) 
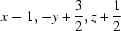
; (v) 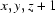.

**Fig. 1.**
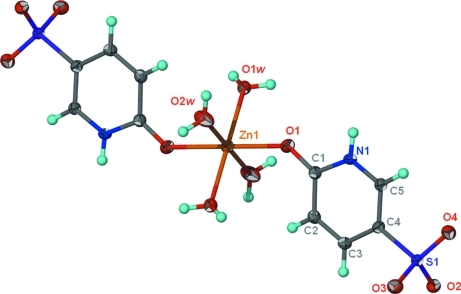
Thermal ellipsoid plot (Barbour, 2001) of Zn(H2O)4(C5H4NO4S)2 at the 50% probability level; hydrogen atoms are drawn as spheres of arbitrary radius.

## supplementary crystallographic information

### Experimental

Zinc carbonate (0.25 g, 2 mmol) was added to a hot aqueous solution of 2-hydroxypyridine 5-sulfonic acid (0.35 g, 2 mmol); the pH value was adjusted to 6 with 0.1 *M* sodium hydroxide. The solution was allowed to evaporate slowly. Colorless prismatic crystals were isolated after five days. CH&N elemental analysis. Calc. for C_10_H_16_N_2_O_12_S_2_Zn:*C* 24.73, H 3.32, N 5.77%; found: C 24.77, H 3.37, N 5.81%.

### Refinement

Carbon-bound H-atoms were placed in calculated positions (C—H 0.93 Å) and were included in the refinement in the riding model approximation, with *U*(H) set to 1.2*U*(C). The ammonium and water H-atoms were refined with a distance restraint of N–H = O–H 0.85±0.01 Å; their temperature factors were refined.

### Crystal data

**Table:**

[Zn(C_5_H_4_NO_4_S)_2_(H_2_O)_4_]	*F*(000) = 496
*M**_r_* = 485.74	*D*_x_ = 1.880 Mg m^−^^3^
Monoclinic, *P*2_1_/*c*	Mo *K*α radiation, λ = 0.71073 Å
Hall symbol: -P 2ybc	Cell parameters from 7685 reflections
*a* = 6.7701 (2) Å	θ = 3.3–27.5°
*b* = 13.9725 (5) Å	µ = 1.74 mm^−^^1^
*c* = 10.0343 (3) Å	*T* = 293 K
β = 115.331 (2)°	Prism, colorles
*V* = 857.93 (5) Å^3^	0.21 × 0.16 × 0.16 mm
*Z* = 2	

### Data collection

**Table:**

Rigaku R-AXIS RAPID IP diffractometer	1951 independent reflections
Radiation source: fine-focus sealed tube	1866 reflections with *I* > 2σ(*I*)
graphite	*R*_int_ = 0.021
ω scans	θ_max_ = 27.5°, θ_min_ = 3.3°
Absorption correction: multi-scan (*ABSCOR*; Higashi, 1995)	*h* = −8→8
*T*_min_ = 0.711, *T*_max_ = 0.768	*k* = −17→18
8224 measured reflections	*l* = −13→13

### Refinement

**Table:**

Refinement on *F*^2^	Primary atom site location: structure-invariant direct methods
Least-squares matrix: full	Secondary atom site location: difference Fourier map
*R*[*F*^2^ > 2σ(*F*^2^)] = 0.023	Hydrogen site location: inferred from neighbouring sites
*wR*(*F*^2^) = 0.066	H atoms treated by a mixture of independent and constrained refinement
*S* = 1.06	*w* = 1/[σ^2^(*F*_o_^2^) + (0.037*P*)^2^ + 0.5079*P*] where *P* = (*F*_o_^2^ + 2*F*_c_^2^)/3
1951 reflections	(Δ/σ)_max_ = 0.001
144 parameters	Δρ_max_ = 0.37 e Å^−^^3^
5 restraints	Δρ_min_ = −0.39 e Å^−^^3^

### Fractional atomic coordinates and isotropic or equivalent isotropic displacement parameters (Å^2^)

**Table:**

	*x*	*y*	*z*	*U*_iso_*/*U*_eq_	
Zn1	0.5000	0.5000	0.5000	0.02207 (10)	
S1	0.81951 (6)	0.69216 (3)	−0.07941 (4)	0.01923 (11)	
O1	0.4186 (2)	0.54472 (10)	0.28771 (14)	0.0295 (3)	
O3	0.9550 (2)	0.77155 (9)	0.00655 (14)	0.0290 (3)	
O2	0.95353 (19)	0.61832 (9)	−0.10431 (13)	0.0264 (3)	
O4	0.63570 (19)	0.72213 (9)	−0.21315 (13)	0.0271 (3)	
O1W	0.20020 (19)	0.43039 (9)	0.40362 (13)	0.0252 (2)	
O2W	0.3496 (2)	0.62596 (11)	0.53828 (17)	0.0406 (3)	
C2	0.7484 (3)	0.55821 (12)	0.25390 (18)	0.0234 (3)	
H2	0.8360	0.5271	0.3413	0.028*	
C1	0.5190 (3)	0.56850 (11)	0.21274 (17)	0.0214 (3)	
N1	0.4007 (2)	0.60683 (10)	0.07675 (15)	0.0229 (3)	
C5	0.4880 (2)	0.64352 (13)	−0.01119 (17)	0.0221 (3)	
H5	0.3979	0.6715	−0.1009	0.027*	
C4	0.7072 (2)	0.63937 (11)	0.03179 (17)	0.0201 (3)	
C3	0.8395 (3)	0.59340 (11)	0.16668 (18)	0.0227 (3)	
H3	0.9891	0.5873	0.1956	0.027*	
H1	0.2625 (16)	0.6084 (16)	0.045 (2)	0.035 (6)*	
H11	0.158 (4)	0.4218 (18)	0.3125 (12)	0.048 (7)*	
H12	0.166 (4)	0.3815 (12)	0.438 (3)	0.045 (7)*	
H21	0.223 (2)	0.6479 (18)	0.509 (3)	0.051 (7)*	
H22	0.431 (4)	0.6538 (17)	0.6168 (18)	0.049 (7)*	

### Atomic displacement parameters (Å^2^)

**Table:**

	*U*^11^	*U*^22^	*U*^33^	*U*^12^	*U*^13^	*U*^23^
Zn1	0.02021 (15)	0.02605 (16)	0.02093 (15)	−0.00103 (9)	0.00973 (11)	0.00145 (9)
S1	0.01744 (18)	0.02194 (19)	0.01890 (19)	−0.00059 (13)	0.00834 (14)	0.00027 (13)
O1	0.0244 (6)	0.0417 (7)	0.0246 (6)	−0.0023 (5)	0.0125 (5)	0.0061 (5)
O3	0.0276 (6)	0.0281 (6)	0.0320 (7)	−0.0085 (5)	0.0133 (5)	−0.0060 (5)
O2	0.0222 (5)	0.0329 (6)	0.0252 (6)	0.0046 (5)	0.0111 (5)	−0.0021 (5)
O4	0.0241 (6)	0.0313 (6)	0.0233 (6)	0.0017 (5)	0.0076 (5)	0.0071 (5)
O1W	0.0249 (6)	0.0286 (6)	0.0223 (6)	−0.0043 (5)	0.0104 (5)	0.0014 (5)
O2W	0.0268 (7)	0.0401 (8)	0.0447 (8)	0.0077 (6)	0.0056 (6)	−0.0145 (6)
C2	0.0214 (7)	0.0268 (8)	0.0198 (7)	0.0036 (6)	0.0069 (6)	0.0030 (6)
C1	0.0223 (7)	0.0218 (7)	0.0204 (7)	−0.0023 (6)	0.0094 (6)	−0.0005 (6)
N1	0.0151 (6)	0.0316 (7)	0.0216 (7)	−0.0009 (5)	0.0074 (5)	0.0019 (5)
C5	0.0203 (7)	0.0264 (8)	0.0190 (7)	0.0007 (6)	0.0079 (6)	0.0024 (5)
C4	0.0198 (7)	0.0215 (7)	0.0204 (7)	−0.0012 (6)	0.0101 (6)	−0.0006 (5)
C3	0.0178 (7)	0.0266 (8)	0.0229 (8)	0.0021 (6)	0.0078 (6)	0.0001 (6)

### Geometric parameters (Å, °)

**Table:**

Zn1—O1^i^	2.0560 (12)	O2W—H21	0.833 (10)
Zn1—O1	2.0560 (12)	O2W—H22	0.838 (10)
Zn1—O1W^i^	2.0788 (12)	C2—C3	1.360 (2)
Zn1—O1W	2.0788 (12)	C2—C1	1.434 (2)
Zn1—O2W	2.1487 (14)	C2—H2	0.9300
Zn1—O2W^i^	2.1487 (14)	C1—N1	1.362 (2)
S1—O4	1.4477 (12)	N1—C5	1.356 (2)
S1—O3	1.4626 (12)	N1—H1	0.850 (10)
S1—O2	1.4643 (12)	C5—C4	1.358 (2)
S1—C4	1.7588 (15)	C5—H5	0.9300
O1—C1	1.2553 (19)	C4—C3	1.418 (2)
O1W—H11	0.841 (10)	C3—H3	0.9300
O1W—H12	0.841 (10)		
			
O1^i^—Zn1—O1	180.0	Zn1—O1W—H12	125.1 (18)
O1^i^—Zn1—O1W^i^	83.37 (5)	H11—O1W—H12	108 (2)
O1—Zn1—O1W^i^	96.63 (5)	Zn1—O2W—H21	137.0 (19)
O1^i^—Zn1—O1W	96.63 (5)	Zn1—O2W—H22	112.2 (18)
O1—Zn1—O1W	83.37 (5)	H21—O2W—H22	109 (3)
O1W^i^—Zn1—O1W	180.00 (6)	C3—C2—C1	120.81 (15)
O1^i^—Zn1—O2W	90.07 (6)	C3—C2—H2	119.6
O1—Zn1—O2W	89.93 (6)	C1—C2—H2	119.6
O1W^i^—Zn1—O2W	88.66 (5)	O1—C1—N1	117.88 (14)
O1W—Zn1—O2W	91.34 (5)	O1—C1—C2	126.76 (15)
O1^i^—Zn1—O2W^i^	89.93 (6)	N1—C1—C2	115.35 (14)
O1—Zn1—O2W^i^	90.07 (6)	C5—N1—C1	124.58 (13)
O1W^i^—Zn1—O2W^i^	91.34 (5)	C5—N1—H1	117.5 (16)
O1W—Zn1—O2W^i^	88.66 (5)	C1—N1—H1	118.0 (16)
O2W—Zn1—O2W^i^	180.0	N1—C5—C4	119.93 (14)
O4—S1—O3	113.63 (8)	N1—C5—H5	120.0
O4—S1—O2	113.28 (7)	C4—C5—H5	120.0
O3—S1—O2	110.91 (7)	C5—C4—C3	118.76 (14)
O4—S1—C4	106.01 (7)	C5—C4—S1	119.40 (12)
O3—S1—C4	106.05 (7)	C3—C4—S1	121.84 (12)
O2—S1—C4	106.28 (7)	C2—C3—C4	120.17 (14)
C1—O1—Zn1	136.67 (11)	C2—C3—H3	119.9
Zn1—O1W—H11	112.6 (17)	C4—C3—H3	119.9
			
O1W^i^—Zn1—O1—C1	28.28 (18)	N1—C5—C4—C3	2.1 (2)
O1W—Zn1—O1—C1	−151.72 (18)	N1—C5—C4—S1	−177.08 (12)
O2W—Zn1—O1—C1	116.92 (17)	O4—S1—C4—C5	−6.85 (15)
O2W^i^—Zn1—O1—C1	−63.08 (17)	O3—S1—C4—C5	114.25 (14)
Zn1—O1—C1—N1	−171.26 (12)	O2—S1—C4—C5	−127.66 (13)
Zn1—O1—C1—C2	9.9 (3)	O4—S1—C4—C3	173.98 (13)
C3—C2—C1—O1	−175.15 (17)	O3—S1—C4—C3	−64.92 (15)
C3—C2—C1—N1	6.0 (2)	O2—S1—C4—C3	53.17 (15)
O1—C1—N1—C5	173.90 (16)	C1—C2—C3—C4	−1.2 (2)
C2—C1—N1—C5	−7.1 (2)	C5—C4—C3—C2	−3.0 (2)
C1—N1—C5—C4	3.2 (3)	S1—C4—C3—C2	176.21 (13)

Symmetry codes: (i) −*x*+1, −*y*+1, −*z*+1.

### Hydrogen-bond geometry (Å, °)

**Table:**

*D*—H···*A*	*D*—H	H···*A*	*D*···*A*	*D*—H···*A*
N1—H1···O2^ii^	0.85 (1)	1.99 (1)	2.790 (2)	157 (2)
O1w—H11···O2^iii^	0.84 (1)	1.98 (1)	2.809 (2)	171 (2)
O1w—H12···O3^iv^	0.84 (1)	1.93 (1)	2.767 (2)	172 (3)
O2w—H21···O3^v^	0.83 (1)	2.13 (1)	2.926 (2)	160 (3)
O2w—H22···O4^vi^	0.84 (1)	1.93 (1)	2.765 (2)	174 (3)

Symmetry codes: (ii) *x*−1, *y*, *z*; (iii) −*x*+1, −*y*+1, −*z*; (iv) −*x*+1, *y*−1/2, −*z*+1/2; (v) *x*−1, −*y*+3/2, *z*+1/2; (vi) *x*, *y*, *z*+1.
